# Experiencing Nature to Satisfy Basic Psychological Needs in Parenting: A Quasi-Experiment in Family Shelters

**DOI:** 10.3390/ijerph17228657

**Published:** 2020-11-21

**Authors:** Elise Peters, Jolanda Maas, Dieuwke Hovinga, Nicole Van den Bogerd, Carlo Schuengel

**Affiliations:** 1 Department of Education, University of Applied Sciences Leiden, 2333 CK Leiden, The Netherlands; hovinga.d@hsleiden.nl (D.H.); n.vanden.bogerd@vu.nl (N.V.d.B.); 2 Department of Clinical, Neuro and Developmental Psychology, Vrije Universiteit Amsterdam, 1081 BT Amsterdam, The Netherlands; Jolanda.maas@vu.nl; 3 Amsterdam Public Health Research Institute, Section of Clinical Child and Family Studies, Vrije Universiteit Amsterdam, 1081 BT Amsterdam, The Netherlands; c.schuengel@vu.nl

**Keywords:** natural environment, parents, shelter, abused women, homeless families, basic psychological needs

## Abstract

Finding fulfillment of basic psychological needs may be difficult for parents living in shelters after becoming homeless or after escaping violence. This study tested if experiencing nature was associated with the basic psychological needs of parents in shelters. Need satisfaction and need frustration were measured among parents in shelters (*N* = 160), with one measurement in the standard indoor context of the shelter and one measurement while experiencing nature. Experiencing nature was associated with enhanced need satisfaction (*d* = 0.28) and reduced need frustration (*d* = −0.24). The effect was especially pronounced for parents with young children. Our findings suggest that the physical environment matters for parents’ basic psychological need fulfillment as they interact with their children in the context of sheltering. This finding opens a potential avenue for supporting parental functioning and resilience in the face of risk if these effects were to be replicated across settings using controlled experimental designs. At the very least, the findings may be discussed with practitioners and parents in the context of making shelter life and work more conducive to mental health and family functioning.

## 1. Introduction

Self-determination theory [1] was conceived to understand the conditions that support or thwart human psychological flourishing. This theory poses three basic psychological needs as essential for growth, integrity and wellbeing: the need for autonomy, the need for relatedness and the need for competence [2]. In the context of parenting, these needs pertain to the freedom to make parenting decisions and to take ownership of one’s own actions (autonomy), feeling close and connected to one’s children (relatedness) and feeling competent and skilled in parenting (competence) [3]. Basic psychological needs have been assessed to understand individual differences in parenting experiences. Parents who find fulfillment of their needs for relatedness, competence and autonomy in their role as parents would be more prone to experience wellbeing [3,4,5,6] and maintain autonomy-supportive parenting practices [5,7,8]. Self-determination theory may therefore be useful for evaluating efforts to optimize the social or physical environment in which parenting occurs.

### 1.1. The Impact of Living in a Shelter on Parents’ Opportunities for Need Fulfillment

While it may sometimes be hard for any parent to find need fulfillment, this may be especially the case for parents who live in shelters after becoming homeless or after escaping violence. Conditions in shelters may limit the possibilities for need fulfillment and may even actively frustrate parents in their attempts. Parents have reported that the crowded, noisy and chaotic living spaces [9,10] and imposed shelter rules [11] limited them in using their own routines and rules [12,13], which may frustrate the need for autonomy. Parents have also reported that the lack of privacy made them vulnerable for scrutiny and criticism by staff and other parents [14], which may frustrate the need to experience competence. Limited access to safe and engaging environments is reported to restrict parents in their opportunities for positive parent–child interactions [15], which may frustrate the need for experiencing relatedness. Moreover, shelter rules and routines may detract from parental authority, roles and responsibilities and, in some cases, even lead to parents stepping away from their parenting duties and ceding this role to shelter staff [12], eliminating satisfaction of basic needs from parenting altogether.

In recognition of the many challenges facing homeless and violence-exposed parents and children, shelters may use several avenues for supporting parents. Shelters provide a safe place to live for parents and their children [16], temporarily support them with practical hassles and stressors [17,18], provide social work to find balance and transition to an independent life [19] and offer specific interventions with regard to, e.g., parent–child relatedness and parental competence [20]. On top of that, shelters may try to enhance the wellbeing of parents and children by introducing nature [21,22,23,24].

### 1.2. Experiencing Nature to Support Parents in Their Need Fulfillment

A qualitative evaluation of the introduction of nature into shelters suggested that experiencing nature may support parents in fulfilling their parental basic needs [25]. In this participatory action research project, professionals observed that having a natural environment at the shelter property allowed parents to undertake activities of their own choice and to interact with their children in ways they deemed fitting, and that being in nature supported parents to feel connected with their child and to have positive ways of interacting. Such immediate and temporary effects of experiencing nature are consistent with the strong day-to-day fluctuations in need fulfillment reported by Brenning, Soenens, Mabbe and Vansteenkiste [3]. It is therefore important to test whether creating more opportunities for experiencing nature would also lead to more need fulfillment.

### 1.3. The Aim of This Study

The primary aim of this study was to investigate if experiencing nature was associated with basic psychological needs of parents in shelters. We expected that parents would report more parental need satisfaction and less parental need frustration when experiencing nature than when they were in the standard indoor shelter environment.

The secondary aim was to test whether children’s age, shelter type and nature connectedness moderated the association between nature and need satisfaction. The theory of affordances [26] suggests that the activities to which physical environments give rise depend on the specifics of the human being in that environment. This means that the support provided by an environment varies from person to person. Parenting roles differ per developmental stage of the child. When children grow older, the demands on the parents change from being close and available towards indirect monitoring and psychological autonomy granting [27]. This means that environments will have specific affordances for parents of younger children (such as allowing or disallowing parents to be monitoring and scaffolding their child while being close by and available) and specific affordances for parents of older children (such as allowing or disallowing parents to support their child in forming their own friendships and to be monitoring the child indirectly). Given the lack of specific theory and previous work suggesting the direction of the moderating effect of a child’s age on the effect of nature on parental need fulfillment, the moderating effect was explored.

Affordances of nature may vary between people who are in a shelter due to homelessness for financial reasons and people who are admitted due to threats of violence. Experiencing nature might be stressful for the latter group because of the risks of being away from the shelter, which affects the balance of threats (or negative affordances [26]) and promises (or positive affordances [26]) from such an environment. For this reason, we include type of shelter as a potential moderator, expecting that the strength of the association between experiencing nature and need fulfillment was strongest for parents who were in a shelter due to homelessness.

Feeling connected to nature may be related to the satisfaction of the basic psychological need of relatedness by allowing feelings of love, kindness and empathy [28]. Although research to date has mainly focused on feelings of relatedness to the world, and not on parent–child relatedness specifically, we do include parents’ nature connectedness as a possible moderator. We expected that the strength of the relation between experiencing nature and need fulfillment was strongest for parents who felt connected to nature.

## 2. Materials and Methods

### 2.1. Participants

This study was conducted among parents with one or more children under the age of 18 who lived with their children in a participating women’s shelter or homeless shelter in The Netherlands at the time of data collection. Parents were excluded from participation when their care professional assessed them not fit for understanding the study information due to illiteracy, language problems and/or intellectual disabilities. The overall majority of participating parents identified themselves as female (91%).

The parents were selected from 20 shelters that participated in a Dutch nationwide project aimed to enhance the wellbeing of families in shelters through the development and use of natural environments. Parents were selected and approached for participation by their shelter care professional. Parents were informed about the goal to study fluctuations in basic psychological need satisfaction and frustration among parents residing in shelters. Parents were explicitly informed that shelter professionals had no access to the provided information and that their participation would have no consequences for the care they and their family received. Parents received no payment.

Based on power analysis in G*Power for two groups, two measurements, with a power of 0.95, alpha of 0.05 and a medium effect of between x within interaction f(V) of 0.10–0.15, we aimed for 146 participants. A total of 167 participants were recruited. Data of seven participants were removed because the data collection did not occur according to procedure, resulting in a total of 160 participants (Table 1).

Data collection took place from October 2018 until February 2019. The Scientific and Ethical Review Board of the Faculty of Behavioral and Movement Sciences of the VU Amsterdam approved of the study protocol (VCWE-2018-0138).

### 2.2. Design and Procedures

This study followed a two (within-subject; measurement) by two (between-subject; environment) crossover quasi-experimental design. Two measurements of parental need satisfaction and need frustration were conducted in two conditions: during the families’ usual daily routine in the standard indoor environment of the shelter, and while the family experienced nature.

Children’s age, shelter type and parents’ connectedness to nature were included as moderating variables.

### 2.3. Intervention

#### 2.3.1. Nature Experience

Shelter care professionals facilitated a nature experience for families under their care. Nature experiences included an experience through sensory perception (e.g., sitting in the sun or listening to bird songs) or through interaction (e.g., gardening or walking the dog) with living organisms like plants and animals, or with—what is in Western cultures called—”non-living” natural elements like water, sunlight and soil. Experiences with nature were personalized based on the professionals’ assessment of the emotional state of the family members (e.g., allowing family members feeling angry to visit a natural place that afforded coping activities), on the families’ current level of risk for being away from the shelter (e.g., allowing families with the highest risk level to experience nature in a protected and enclosed natural space and allowing more freedom of movement for families with lower risk levels) and on the religious and cultural backgrounds of the family (e.g., allowing Muslim families to experience only halal nature experiences). We chose for this personalization to make inclusion of the very diverse population of shelter clients possible. Professionals chose a nature experience based on their general knowledge of the family and which was responsive to the family’s possibilities and needs. Professionals initiated the nature experience and were present when the family experienced nature.

#### 2.3.2. Comparison Condition

Shelter care professionals visited the families for their usual daily check-in with the family during a moment of parent–child interaction in the family’s daily routine in the standard indoor environment of the shelter.

### 2.4. Data Collection

The research protocol defined a three-week period for data collection. Each professional chose a moment within these three weeks to deliver the intervention to the family. Professionals chose a moment for delivering the comparison condition within a seven-day time span from the intervention.

During the nature experience and in the comparison condition, parents filled out an online questionnaire about their own age and gender and that of their child, their parental need satisfaction and need frustration and their connectedness to nature. When parents were not able to read the questionnaire independently, the professional sat opposite of the parent, read the questions and possible answers out loud and allowed the parents to answer the questions privately.

At both measurements, professionals filled out an online questionnaire in which they provided the date and time, the name of the shelter, a written observation of the need of the parent based on the question “What parental need did the parent have at this moment?”, a written description of the activity based on the question: “What exactly happened? Describe the activity”, and a written observation based on the questions: “What did you notice in the parent? And what else? And what else?”. In this study, we used the observational data only for checking if the intervention met the criteria of an experience through a sensory perception or interaction with living organisms or “non-living” natural elements.

To be able to check for sequence effect, the shelters were manually preassigned to two pre-specified subgroups, aiming for two subgroups of the same size and with an equal division of women’s shelters and homeless shelters. Participants from shelters in subgroup one (*N* = 92) did the standard indoor environment of the shelter (comparison condition) first and the nature experience (intervention) second. For participants from shelters in subgroup two (*N* = 68), this sequence was reversed.

### 2.5. Measurements

#### 2.5.1. Psychological Need Satisfaction and Frustration

The twelve questions from the Dutch parenting version [3,7] of the validated Basic Psychological Need Satisfaction and Need Frustration Scale [29] were used to assess psychological need satisfaction and frustration. The questionnaire contains statements on satisfaction of the basic psychological need of relatedness (e.g., “Today, I felt connected with my child”), competence (e.g., “Today, I felt confident in what I did for my child”) and autonomy (e.g., “Today, I felt a sense of choice and freedom in the things I did with my child”), as well as statements on the frustration of basic needs of relatedness (e.g., “Today, I felt a distance between my child and me”), competence (“Today, I felt insecure about my abilities with my child”) and autonomy (e.g., “Today, I felt forced to do things for my child I did not choose to do”). Items were rated on a scale from 1 (completely not true) to 5 (completely true). Average scores were created by computing the average of the six items for need satisfaction and the six items for need frustration. The Cronbach’s alphas for need satisfaction and need frustration were both 0.74.

#### 2.5.2. Connectedness to Nature

Schultz’s [30] Inclusion of Nature in Self Scale (INS) was used to assess connectedness with nature in both parents and professionals. This graphical single-item scale contains seven pictures of two circles, with one circle named “self” and the other circle named “nature”, which differ in degree of overlap. Parents and professionals were asked to rate their connectedness to nature by choosing one of the seven pairs of circles. Circle pairs were rated from 1 (complete separation of the two circles) to 7 (complete connection of the two circles). Although Martin and Czellar [31] suggested an extension on INS to improve the construct and predictive validity, the single-item INS showed a workable test–retest reliability of 0.77 (*p* < 0.001) [31] and was chosen because it is concise and easy to administer. For analyses, we used the average between the INS score measured in the indoor context and the INS score measured while experiencing nature.

#### 2.5.3. Children’s Age and Shelter Type

Children’s age in years was reported by the parent. Shelter type (being a shelter for homeless families, a women’s shelter or a combined women’s and homeless shelter) was reported by the parent’s care professional.

We refrained from collecting other personal data to limit the amount of identifiable information.

### 2.6. Quality of Measurements

#### 2.6.1. Training

Professionals were trained in four training sessions to be able to facilitate a nature experience for families in their care and to be able to collect data according to the research protocol. After the first training session, the professionals conducted a tryout of data collection in which they got feedback regarding the consistency with the research protocol. After the second and third training session, data collection occurred. The fourth training session was a closing session with reflection on the results of the study.

#### 2.6.2. Setting Conditions

To allow the participation of parents with diverse backgrounds and safety concerns, it was necessary that all shelters had safe natural environments on their own property. Each shelter received funding for developing a natural environment, varying from EUR 10,000 (approximately USD 11,080) to EUR 65,000 (approximately USD 72,000). Shelters developed a restorative garden, a natural play area, a children’s farm or a vegetable garden. Shelters were assisted in the development of the natural environments by spatial planners, animal experts, gardeners and construction workers. Data collection started when all shelters had the possibility to use a natural environment. The natural environments that were used in this study are specified in Table A1.

#### 2.6.3. Statistical Analyses

The associations between experiencing nature and parental need frustration and need satisfaction were analyzed using linear mixed model analyses in SPSS Statistics for Windows, version 27 (SPSS Inc., Chicago, IL, USA), accounting for the clustering of the two measurements (level 1) within participants (level 2) within shelter locations (level 3). Although we were not interested in the higher-order effects, we chose to incorporate the shelter location as level 3 to be able to produce more accurate standard errors [32]. The unstandardized coefficients were converted to standardized mean differences (Cohen’s d) [33]. Analyses of effect modification using two-way interaction terms were conducted for each of the potential moderators. Interaction terms with a *p*-value lower than 0.05 were identified as moderators.

## 3. Results

### 3.1. Participant Flow

**Figure 1:**
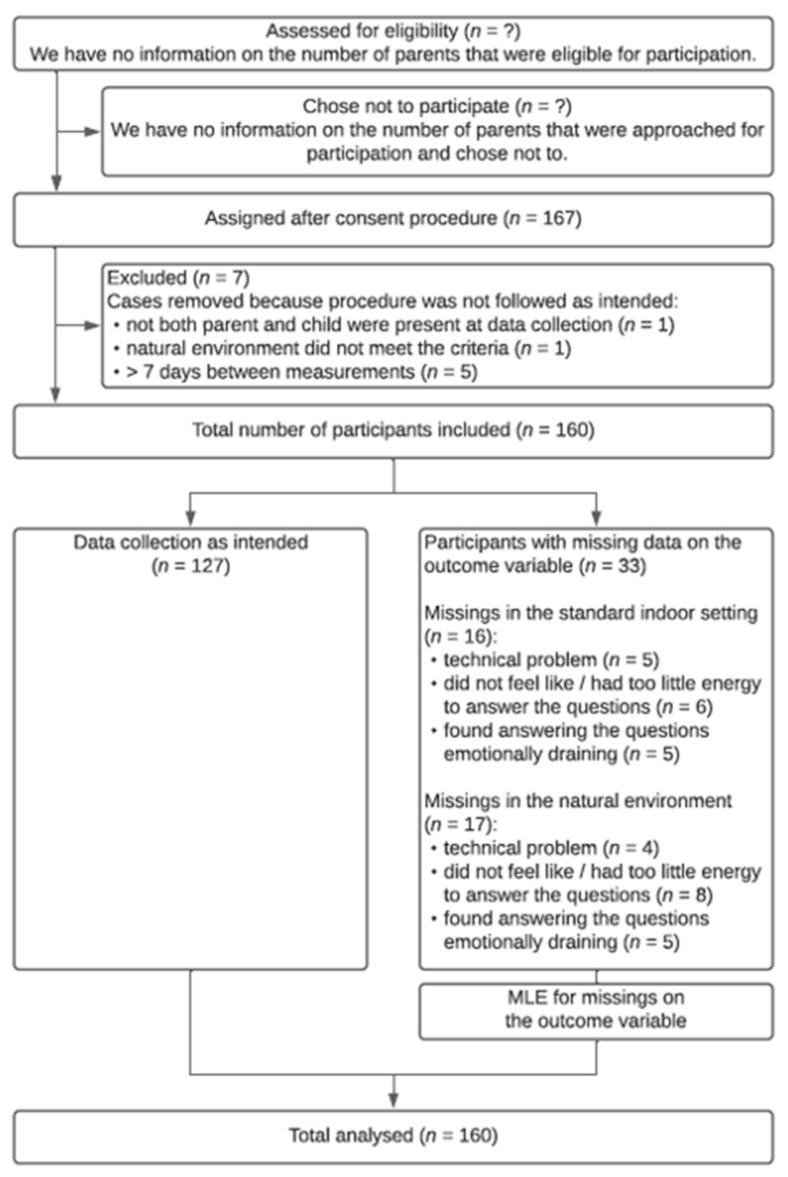
The flow of participants with the total number of participants in each group at each stage, and reasons for drop out.

The participant flow with the total number of participants at each stage of the study is given in Figure 1, including reasons for drop out. We computed maximum likelihood estimates for missing data on the outcome variable.

Table 1 shows the characteristics of the study population.

**Figure A1:**
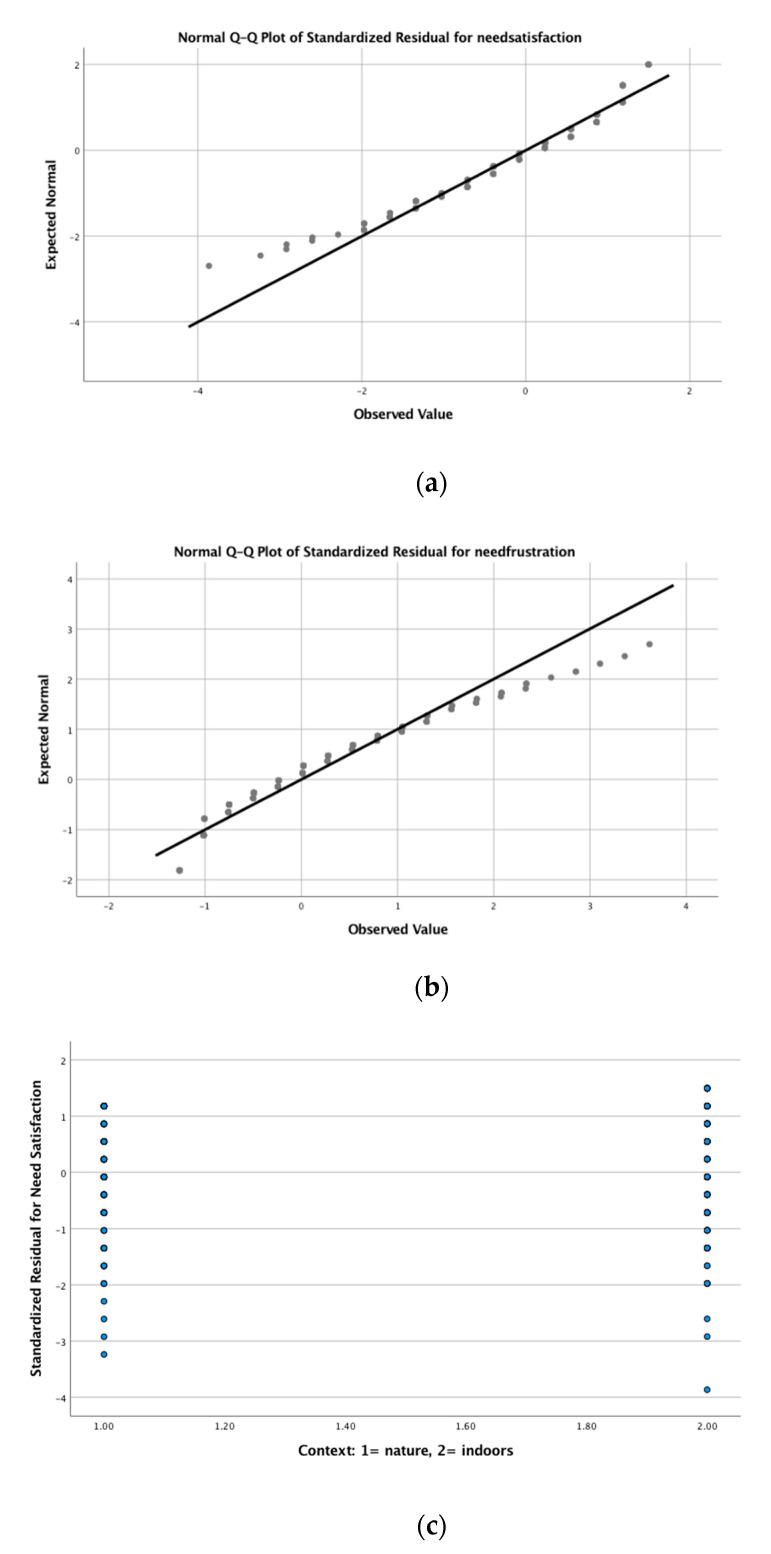

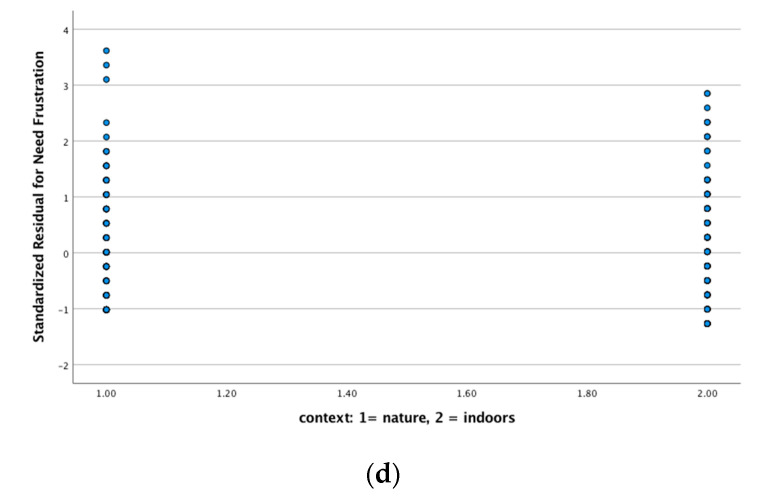
Q–Q Plots of the Standardized Residuals, and Scatter Plots of the Variation of Standardized Residuals for the Two Contexts. (**a**) Q–Q Plot of the Standardized Residuals for Need Satisfaction. (**b**) Q–Q Plot of the Standardized Residuals for Need Frustration. (**c**) Scatter Plot of the Variation of Standardized Residuals for Need Satisfaction per context. (**d**) Scatter Plot of the Variation of Standardized Residuals for Need Frustration per context.

The normal distribution of the residuals was moderately skewed (for need satisfaction −0.75 (SE = 0.15), for need frustration 0.94 (SE = 0.14)) and had low kurtosis (for need satisfaction 0.5 (SE = 0.29), for need frustration 0.59 (SE = 0.29)). Q–Q plots and scatter plots showed a proximal normal distribution (Figure A1).

**Figure 2:**
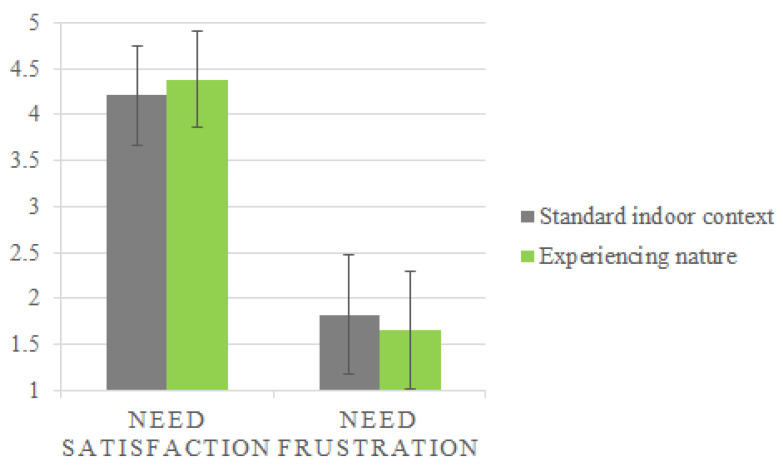
Means with error bars (1SD) for need satisfaction and need frustration in the standard indoor context and while experiencing nature.

Figure 2 shows the basic statistics on parental need satisfaction and need frustration. Participants reported a higher parental need satisfaction (Mnature = 4.38, SD = 0.52, 95% CI 4.29–4.46/Mindoor = 4,21, SD = 0.54, 95% CI 4.11–4.3) and a lower parental need frustration (Mnature = 1.66, SD = 0.64, 95% CI 1.55–1.77/Mindoor = 1.82, SD = 0.65, 95% CI 1.71–1.93) while experiencing nature compared to being in the standard indoor environment of the shelter.

### 3.2. Associations between Experiencing Nature and Parental Self-Determination

Multilevel regression analyses showed that parents reported statistically significant higher scores on need satisfaction and statistically significant lower scores on need frustration when experiencing nature as opposed to being in the indoor environment (Table 2).

Interaction terms (see Table A2) for the moderating effect of sequence, type of shelter and parents’ connectedness to nature were not statistically significant. The interaction term for child age, however, was (*p* = 0.01 for need satisfaction, *p* = 0.02 for need frustration). The difference between basic psychological needs while experiencing nature as opposed to being in the standard indoor environment was bigger for participants with younger children (for need satisfaction B 0.04, SE 0.01, 95% CI 0.01–0.07; for need frustration B −0.04, SE 0.02, 95% CI −0.08–−0.01).

## 4. Discussion

Having parents in shelters experience nature was associated with higher parental need satisfaction (*d* = 0.28) and lower parental need frustration (*d* = −0.24). This association was especially pronounced for parents with young children.

When comparing the effect size to other self-determination-informed interventions (see Ntoumanis et al. [34]), the effect of experiencing nature was small. The intervention can, however, be considered promising as the study shows that a single nature experience is associated with improved basic psychological needs. Razani et al. [35] suggested that repeated visits to natural environments were necessary for a maximal effect on parental wellbeing. Future studies could investigate if more regular nature experiences could further enhance the effect size. Furthermore, the small effect can be considered as promising because the intervention was directed “only” to the experience of nature. Future studies may combine experiences in nature with other self-determination theory-informed interventions such as goal setting or social support (see e.g., Ntoumanis, Ng, Prestwich, Quested, Hancox, Thøgersen-Ntoumani, Deci, Ryan, Lonsdale and Williams [34]) to see if such combination increases the effect size.

The sample that we studied mainly consisted of mothers (91%), as is to be expected with the majority of the participating shelters being women’s shelters focusing primarily on female clients. Previous studies have shown that gender affects the relationship between nature experiences and outcome measures. For women, effects of nature experiences were smaller for depressive mood [36] and perceived stress [35], and larger for perceived quality of life [37], levels of activity [38] and self-reported well-being [38] than for men. Future studies should include men in shelters to identify the role of gender on the relationship between nature experiences and basic psychological needs.

Transactional-ecological models [39] show that parents and children are part of ecological settings that change and are changed by the participants in them in complex interactive processes. Germane to the interpretation of the intervention effect is that the change in physical environment was not only a change in environment in which interactions took place, but that the change in scenery changed the actors (parents, children and professionals) and the interactions between them in a complex manner. These complex interactions make it difficult to understand the pathways between experiencing nature and parental basic needs. An example of a possible pathway is through parents’ stronger feelings of affect [40] and vitality [35,41], and lower depressive feelings [42] in nature, which are aspects that Brenning, Soenens, Mabbe and Vansteenkiste [3] showed to correlate with parental basic need fulfillment. Another example of a possible pathway is through nature as an interesting play area for children [43] that provides a wide range of play possibilities [44,45] and in turn allows a range of child behaviors with also room for loud, active or even destructive behavior that parents can otherwise experience as negative child behavior. Not having to evaluate the child’s behavior as negative may strengthen parents’ feelings of relatedness, competence and autonomy. A third possible pathway is that shelter professionals themselves benefited from the restorative qualities of nature, resulting in, e.g., stress reduction [46], positive emotions [47] or attention restoration [48], which could have changed their professional interactions with the family and so impacted parents’ basic psychological need fulfillment. Future research can contribute to forming informed hypotheses on the interactive processes involved.

The association between experiencing nature and parental basic psychological needs was stronger for parents of younger children than for parents of older children. This raises the question whether the natural environments in this research were suitable to support parent–child interactions with children of all ages. When children in higher age groups develop towards self-confidence, peer group membership and autonomy, the demands on the parent changes from being close and available towards indirect monitoring and psychological autonomy granting [27]. The majority of the available natural environments were relatively small and confined (such as a courtyard garden), which were likely not fitting for parent–child interactions with older children.

Contrary to our hypothesis, associations between need frustration and satisfaction and context were not significantly moderated by shelter type. It remains, therefore, unclear to what extent families seeking shelter for acute safety or families who are homeless benefit differently. The fact that professionals chose a nature experience responsive to the families’ possibilities and needs may have prevented parents from women’s shelters to experience limited promises and larger threats due to their safety issues. This finding may motivate professionals to use nature experiences for parents with safety concerns, given that they do this whilst being responsive to the families’ possibilities and needs.

Additionally, contrary to our hypothesis, associations between experiencing nature and need frustration and need satisfaction were not significantly moderated by the parent’s nature connectedness. It remains, therefore, unclear to what extent parents with higher or lower nature connectedness benefit differently. This may motivate professionals to use nature experiences for parents with low connectedness to nature just as well as for parents with high connectedness to nature. For the interpretation of this finding, we must consider the low response rate (*N* = 65), which gives reason to be cautious with interpretation. The low response to this question could be due to the fact that the question was the last of the questionnaire and came directly after the Basic Psychological Need Satisfaction and Need Frustration Scale which professionals reported as challenging on the concentration and emotion of the parents. We advise future research to schedule the INS questionnaire at a separate time point.

For the interpretation of the findings, it is important to note that all data were collected during the fall and winter seasons, which were relatively cold and dark months (with an average temperature of seven degrees Celsius, and nine hours between sunrise and sunset), with relatively dry weather (with an average of 51 mm of rain per month) [49]. Although several studies have suggested that weather conditions can impact the restorative qualities of nature experiences [50,51], little is known about the impact of weather on the restorative qualities of nature for parental need satisfaction specifically. Future studies may use a variety of weather conditions to identify if and to what extent these can impact the results.

### Sources of Potential Bias

The selection of participants is a potential weakness in the study design. Firstly, it is possible that selection bias occurred with professionals selecting the parents that they expected to benefit from a nature experience. Secondly, we did not have information regarding the number and characteristics of parents who were eligible for participation but not approached, nor the number and characteristics of parents that dropped out in the informed consent procedure, which makes it impossible to assess if parents who participated differed from eligible participants. This forms a threat to the generalizability of the study results.

This study used a variety of natural environments. Although studies have reported on physical characteristics that make a natural environment higher or lower in quality for different outcome measures [42,43,44] and suggestions have been made for the design of natural environments in care facilities specifically [52,53,54], little is known on the physical characteristics of natural environments for supporting parental basic psychological needs. This lack of insight in supportive physical characteristics for parental basic psychological needs makes it difficult to assess and reflect on the quality of nature used in this study. Future research should focus on identifying physical characteristics of environments that support parental needs, to help future study design as well as practice.

This study used a variety of nature experiences, individualized for each parent and their family. Generalizing the study results to nature experiences of other parents must be done with caution.

This study is a field experiment in a natural setting, which gives ecological validity as well as limitations in the number of variables under control by the researcher. Future research should use more controlled research designs.

## 5. Conclusions

Findings suggest that the physical environment matters for parents’ basic psychological need fulfillment as they interact with their children in the context of sheltering. This finding opens a potential avenue for supporting parental functioning and resilience in the face of risk if these effects were to be replicated across settings using controlled experimental designs. At the very least, the findings may be discussed with practitioners and parents in the context of making shelter life and work more conducive to mental health and family functioning.

## Figures and Tables

**Table 1:** Characteristics of the study population: continuous variables presented as means with standard deviations (SD); categorical variables as numbers (n) with percentages (%).

Variable	N (%)	Mean (SD)	Range	Missing
Shelter type				
-Women’s shelter	112 (70%)			
-Shelter for homeless families	29 (18%)			
-Combined women’s/homeless shelter	19 (12%)			
Age of parent		32 (6.9)	19–65	26 (16%)
Gender of parent				10 (6%)
-Female	145 (91%)			
-Male	1 (<1%)			
-X (third gender or no gender)	4 (3%)			
Parent’s nature connectedness		4.12 (1.6)	1–7	95 (59%)
Child’s age		5.28 (3.6)	0–16	37 (23%)

**Table 2:** Multilevel regression of the association between experiencing nature as opposed to being in the indoor context and parental need satisfaction and parental need frustration; regression coefficient B with 95% confidence intervals, converted to Cohen’s d with 95% CI.

**Measurement**	**Context**	**B (SE)**	**95% CI**	***d*** **(95% CI)**
Need satisfaction				
	Standard indoor context (ref)			
	Experiencing nature	0.18 (0.05)	(0.09–0.27) ***	0.28 (0.14–0.43)
Need frustration				
	Standard indoor context (ref)			
	Experiencing nature	−0.18 (0.06)	−0.3–−0.07) **	−0.24 (−0.4–−0.09)

** *p* < 0.01 *** *p* < 0.001.

## Appendix A

**Table A1:** Natural Environments Used for Experiencing Nature.

Environment	*N* Cases
Garden on the shelter property	48
Neighborhood green	35
Children’s farm	24
Park	22
Indoor nature (e.g., visiting pets in the shelter living space, an interior garden)	11
Natural playground	10
Forest	9
Beach	1

**Table A2:** Potential Effect Modifiers: Estimates of Fixed Effects with Standard Error, 95% Confidence Interval and Significance.

Effect Modifiers Tested	Need Satisfaction			Need Frustration		
	B (SE)	95% CI	Sig.	B (SE)	95% CI	Sig.
Standard indoor environment (Ref)						
Experiencing nature	0.12 (0.15)	−0.18–0.42	0.42	−0.17 (0.19)	−0.55–0.21	0.38
Sequence effect	0.23 (0.16)	−0.1–0.56	0.17	−0.015 (0.21)	−0.43–0.4	0.94
Sequence effect interaction term	−0.04 (0.09)	−0.22–0.14	0.68	0.01 (0.12)	−0.22–0.24	0.94
Standard indoor environment (Ref)						
Experiencing nature	0.16 (0.11)	−0.07–0.38	0.16	−0.13 (0.15)	−0.42–0.15	0.36
Type of shelter	0.12 (0.13)	−0.13–0.37	0.34	−0.15 (0.17)	−0.47–0.18	0.38
Type of shelter interaction term	−0.02 (0.07)	−0.16–0.12	0.82	0.03 (0.09)	−0.15–0.21	0.72
Standard indoor environment (Ref)						
Experiencing nature	0.36 (0.09)	0.18–0.54	0.00	−0.42 (0.12)	−0.65–−0.19	0.00
Age of the child	−0.08 (0.02)	−0.13–−0.03	0.00	0.09 (0.03)	0.03–0.15	0.00
Age of the child interaction term	0.04 (0.01)	0.01–0.07	0.01 *	−0.04 (0.02)	−0.08–−0.01	0.02 *
Standard indoor environment (Ref)						
Experiencing nature	0.09 (0.16)	−0.24–0.41	0.6	−0.48 (0.24)	−0.95–−0.01	0.05
The parent’s connectedness to nature	0.14 (0.07)	0.01–0.28	0.03	−0.23 (0.09)	−0.2–0.16	0.79
The parent’s connectedness to nature interaction term	−0.02 (0.04)	−0.1–0.05	0.51	−0.06 (0.05)	−0.16–0.05	0.29

* *p* < 0.05.
